# Ghost Management: How Much of the Medical Literature Is Shaped Behind the Scenes by the Pharmaceutical Industry?

**DOI:** 10.1371/journal.pmed.0040286

**Published:** 2007-09-25

**Authors:** Sergio Sismondo

## Abstract

Sismondo discusses how pharmaceutical companies and their agents shape multiple steps in the research, analysis, writing, and publication of articles.

## From Ghost Writing to Ghost Management

“What is the purpose of publications?…[The] purpose of data is to support, directly or indirectly, the marketing of our product.” [1]

There are many reports of medical journal articles being researched and written by or on behalf of pharmaceutical companies, and then published under the name of academics who had played little role earlier in the research and writing process [2–14]. In extreme cases, drug companies pay for trials by contract research organizations (CROs), analyze the data in-house, have professionals write manuscripts, ask academics to serve as authors of those manuscripts, and pay communication companies to shepherd them through publication in the best journals. The resulting articles affect the conclusions found in the medical literature, and are used in promoting drugs to doctors.

For example, as reported in *The New York Times* [4], an *Annals of Internal Medicine* article on Merck's “Advantage” trial of Vioxx omitted some trial participants' deaths. Distancing himself from the *Annals* article, first author Jeffrey Lisse said in an interview that “Merck designed the trial, paid for the trial, ran the trial…Merck came to me after the study was completed and said, ‘We want your help to work on the paper.’ The initial paper was written at Merck, and then it was sent to me for editing” [4].

Such incidents have provoked many commentaries about ghost writing in the medical press. This article enlarges the focus from ghost *writing* to the more general ghost *management* of medical research and publishing: when pharmaceutical companies and their agents control or shape multiple steps in the research, analysis, writing, and publication of articles. Such articles are “ghostly” because signs of their actual production are largely invisible—academic authors whose names appear at the tops of ghost-managed articles give corporate research a veneer of independence and credibility. They are “managed” because those companies shape the eventual message conveyed by the article or by a suite of articles. As discussed below, a substantial percentage of medical journal articles (in addition to meeting presentations and other forms of publication, which are not the focus here) are ghost managed, allowing the pharmaceutical industry considerable influence on medical research, and making that research a vehicle for marketing.

Ghost writing and honorary authorship are not in and of themselves scientific problems, though they become so when they shape science to meet particular interests [1]. Some honorary authors are senior professors and chairs of departments, who are added to articles because of local academic politics rather than at the request of drug companies [15,16]. Some busy independent research units hire writers to improve manuscripts; Max Lagnado has argued that professional medical writers can “benefit the scientific community when used in a responsible manner” [15]. In any case, the writing of a manuscript may not be the key point at which behind-the-scenes influence is exerted: study design, statistical analysis, or the choice of placement of manuscripts may be equally important.

It has been repeatedly and firmly established that pharmaceutical company funding strongly biases published results in favor of the company's products [17–19]. Ghost management amplifies that bias, because when one set of commercial interests exerts influence at multiple stages of research, writing, and publication, it will shape the resulting article. In turn, bias affects medical opinion and practice, and ultimately, patients.

## How Common Is Ghost Management?

Because ghost management is hidden, we cannot tell how common it is from published exposés. Current practices in the medical sciences legitimately allow people to serve as authors on the basis of narrow contributions. Therefore many near-honorary authors find little reason to feel uncomfortable with their roles. Fully honorary authors may not see enough of the process of the production of their articles to know that they are ghost managed. Finally, it is not in the interests of writers, authors, or sponsors and their agents to reveal ghost management processes; hence a number of the published accounts of ghost management have stemmed from legal proceedings and investigative journalism. So how common is ghost management?

Much of the information on ghost writing does not help to answer this question. Surveys to quantify rates of ghost writing do not address the ghost management phenomenon, because management may not involve writing, and writing may not be managed [20,21]. However, information about ghost authors, people who should be receiving author credit, strongly suggests that ghost management is common. A study comparing protocols and corresponding publications for industry-initiated trials approved by the Scientific-Ethical Committees for Copenhagen and Frederiksberg in 1994–1995 found evidence of ghost authorship in 75% of these publications (95% confidence intervals, 60%–87%) [22]. Company statisticians were common unacknowledged contributors, but so were the creators of trial designs and protocols, and the writers of manuscripts. The study also found that most (172 of 274) trials for which protocols had been submitted were never begun, completed, or published.

## A Benchmark Study

The most solid information available on ghost management comes from the work of David Healy and Dinah Cattell. A lawsuit in which Healy was involved allowed access to a document listing 85 manuscripts on sertraline that were being coordinated for Pfizer by the medical education and communication company (MECC) Current Medical Directions (CMD) [9,23]. The document lists other agencies as the vendors of some of the documents, and some authors “TBD” (to be determined), so it is almost certain that a number of these 85 manuscripts were written by professional writers acting for Pfizer, possibly via CMD.

More importantly, all of the manuscripts were being managed very carefully, as CMD was aware of submission dates, journals' requests for revisions, target dates for those revisions, and projected publication dates. Authors were not acting independently. The document is peppered with comments such as “First draft with author for review,” and “Manuscript submitted to American Journal of Psychiatry 7/98. Confidence intervals requested by journal. Revised manuscript resubmitted 9/98” [24]. Most manuscripts were published in prestigious medical journals between 1998 and 2000, with academic researchers listed as their authors. The resulting articles are Pfizer's early contribution on the literature on sertraline, but bear few marks of Pfizer's influence, let alone disclosure of Pfizer's contributions to multiple stages of their research, writing, and submission.

These 85 manuscripts became a significant portion of all of the articles published on sertraline. A general Medline search of 1998 to 2000 (performed December 2006) found 479 results with the keyword “sertraline,” and 211 with “sertraline” in the title. This suggests that between 18% and 40% of articles on sertraline in this key period were managed by Pfizer through this one MECC, large enough percentages to have substantial effects on the overall shape of the medical literature on this drug.

Healy and Cattell claim that the CMD articles are uniformly positive about sertraline, and they note under-reporting of side effects in these articles. Compared to other articles on sertraline (i.e., those not coordinated by CMD), the CMD articles were published in more prominent journals, had nearly twice as many authors per article, had authors who were on average twice as prolific, and garnered nearly three times as many citations (20.2 versus 7.7 in Healy and Cattell's analysis) [23]. Apparently, CMD was effective at helping publish these articles in a visible way.

While we cannot know all of the ways in which the CMD document represents other publication efforts, there is strong evidence that ghost management of medical research is common and is part of campaigns by pharmaceutical companies to publish favorable results and key marketing messages.

## MECCs and Ghost Management: A Supply-Side Analysis

A survey in 2001 identified 182 MECCs in the United States, up from 153 in 1998 [25]. A number specialize in producing, placing, and tracking journal articles, known in the trade as “publication planning” or “strategic communication planning.” While these firms hide details of their work—from potential critics and competitors—they also energetically promote themselves and their services. Many have flashy Web sites highlighting their ability to prepare meeting presentations and publish articles.

In preparing this article, I spent six hours searching web pages for MECCs offering publication planning or similar or overlapping services to the pharmaceutical industry, and found 23 (list available from the author). This is not an estimate of the number of such firms, but indicates how common they are. There may be many more firms providing publication planning, including some not uncovered in this search, and some not advertising these services on the Internet. For example, CMD was not among the 23 found, as its current Web site lists only medical education and meeting services as core capabilities. Pharmaceutical companies also do publication planning in-house, though one industry source estimates that in-house planning makes up only 20% of this business [26]. On the other side, it is possible that some of the identified firms misrepresent themselves, and perform only minimal publication planning.

Pharmaceutical companies control an immense quantity of data. The industry provides twice as much funding for clinical trials and related research as do not-for-profit agencies [27]. Of industry funding, 70% goes to CROs that neither make ownership claims on data nor expect to publish the data themselves: CROs perform research to order [28]. By its nature CRO research tends to be ghostly. The 30% of industry funding that goes to academic researchers often also comes with strings attached that can allow sponsors to prepare drafts, edit drafts, delay publication, prevent full access to data, and so on—in short, creating conditions that allow for ghost management [29–31].

In a primer on publication planning, the director of one MECC defines the activity as: “gaining product adoption and usage through the systematic, planned dissemination of key messages and data to appropriate target audiences at the optimum time using the most effective communication channels” [32]. These channels are such things as: “publications, journal reviews, symposia, workshops, advisory boards, abstracts, educational materials/PR.” Influencing scientific opinion in the service of marketing is the clearly stated goal here. The author of this article therefore makes scientific and commercial goals equal stakeholders in communication: in a chart he juxtaposes “Where shall we publish this study?” with “Who are our customers?” and “What can we claim from the results?” with “What are our customer needs?”

Complete Healthcare Communications (CHC) claims on its banner that it “has honed the systems and skills needed to develop the intellectual heart of pharmaceutical marketing—the publication plan. The result for your product? A continuum of awareness, interest, and prescriber confidence” [33]. CHC will manage article submissions to meetings, and as samples of its service it provides hypothetical lists of abstracts and presentations, with their status, dates of presentation, etc. On its Web site is a list of ten hypothetical trials and at least 24 articles that can be written from them, which will lead to a completed bibliography of publications [34].

CHC includes among its clients Pfizer, Sanofi-Aventis, Ortho Biotech, Wyeth, Schering-Plough, Shire, AstraZeneca, and other pharmaceutical companies. It provides testimonials from sponsors and authors. A Johns Hopkins author writes “Very nice outline! You guys are quite organized!! I think it's superb. Very fair and balanced. I'm not used to working with such excellent writers!” CHC claims to have written and submitted over 500 manuscripts, with an acceptance rate of 80%. CHC is able to achieve such a rate with resources far beyond the reach of most researchers: not only are all of its studies fully supported by the largest of pharmaceutical companies, but it boasts a team of 40 medical writers, editors, and librarians.

Other agencies offer very similar services. As described in an article by three of its managers, the Medical Knowledge Group starts publication planning with a phase of exploring “key messages” and “author/journal options” before designing any publications to incorporate those messages [35]. It then tracks those and competitors' messages using its own information management tool. (Like CMD, the Medical Knowledge Group was not included when I conducted my web search, underscoring the limitations of that search.) Another MECC, Envision Pharma, says that “data generated from clinical trials programs are the most powerful marketing tools available to a pharmaceutical company.” Envision will work from early on in the process to ensure “consistent message dissemination,” will plan and track the “data dissemination plan,” and will produce “scientifically accurate, commercially focused abstracts, posters, and primary and secondary publications” [36].

In addition to the publication planners, a much higher number of medical writing companies and individual writers create articles and presentations without engaging in broader publication planning; these may be adjuncts to publication planners. To provide an indication of the scale, the American Medical Writers Association boasts a membership of more than 5,000 [37]; judging from the organization's officers and the content of its conferences, it appears to be dominated by MECCs [38,39].

Several of the publication planning firms identified are owned by major publishing houses. For example, Excerpta Medica is “an Elsevier business” and writes that its “relationship with Elsevier allows… access to editors and editorial boards who provide professional advice and deep opinion leader networks” [40]. Wolters Kluwer Health draws attention to its publisher Lippincott Williams & Wilkins, with “nearly 275 periodicals and 1,500 books in more than 100 disciplines,” and to Ovid and its other medical information providers, emphasizing the links it can make between its different arms [41]. Vertical integration is attractive in the industry as a whole: at least three of the world's largest advertising agencies own not only MECCs, but also CROs [13].

Ghost management of medical journal publications is clearly a substantial business, employing thousands of marketers, writers, and managers. It is large enough that the industry has established the International Publication Planning Association. This organization, which appears to be dominated by pharmaceutical companies, organizes meetings, keeps a directory of experts, and gives awards to honor planners [42]. In addition, the International Society for Medical Publication Professionals also organizes meetings, has committees to develop policy, and posts job advertisements [43]. Both of these associations compete with for-profit companies offering similar services, such as the Center for Business Intelligence, which held forums for Strategic Publication Planning in 2005 and 2006 [44].

## Discussion

Merck's ghost management of the Advantage trial paper was described as “an unusual practice” when it was reported in *The New York Times* [4]. Given the amount of data that pharmaceutical companies control, the number of publication planning agencies that openly advertise on the Internet, the number of medical writers, the existence of two associations for publication planners, and meetings organized and reports written for them, we can conclude that ghost management is common. The CMD document obtained by Healy suggests that during key marketing periods as many as 40% of published articles focusing on specific drugs are ghost managed [24]. Even if the more typical figure is half that, ghost management exerts a huge force on the shape of scientific opinion on new drugs, and does so in the service of marketing.

Articles in medical journals have real effects upon physician prescribing behavior, which is why pharmaceutical companies invest so much in their publication. Journal articles are heavily used in detailing, to validate claims and rebut worries. Even independent of detailers, responsible physicians and medical researchers search the literature to gather evidence about the best treatments. Published scientific articles are the sources of medical information with the highest authority. Systematic reviews and meta-analyses almost all start with the published literature—so even fully independent reviews are influenced by ghostly activities. Therefore, the ghost management of journal articles is a step in the intervention into medical practice.

There are no straightforward solutions, short of large changes to the nature of medical publishing and/or research, changes that would effectively sequester pharmaceutical company funding from research and publishing [45] or from marketing [46]. Until such changes come about, at least we can hope for more awareness of and responsiveness to the issue.

Peer review has not been proven to be an effective tool for quality control, so we cannot rely on journals' peer review systems to guard against biases created by ghost managing [47–49]. Indeed, MECCs are effective at creating publishable articles and getting them published in peer-reviewed journals. Nor are current disclosure measures effective. Major journals have put in place strong disclosure procedures, but while these might disallow extreme forms of ghost management, many forms of it do not run afoul of any rules other than failure to acknowledge some contributors and facilitators. With awareness of the issue, however, perhaps journal editors can recognize signs of behind-the-scenes work. They can refuse to deal directly with publication planners, and they can ask authors repeatedly about under-recognized and over-recognized contributors, facilitators, and influences; systematic adoption of a strong “film credit model” of authorship, in which authors rigidly and closely specify their roles, might aid in those efforts [50]. Such efforts would have to go hand-in-hand with penalties for misconduct [12]. Although not discussed here, MECCs ghost manage other forms of publication including academic meeting presentations, and thus program committees of these meetings face similar issues.

Universities and academic health centers should prohibit contracts that allow sponsors to draft, edit, or suppress articles, or that allow sponsors to keep data from authors; they should even prohibit sponsors from facilitating publication. Universities should also take disciplinary action against investigators who serve as authors on ghost-managed articles. Meanwhile, investigators need to be aware of the mechanisms of ghost management of work that goes under their names, and to refuse to participate. Perhaps they need to be more modest about how many articles they can publish, and more realistic about the amount of effort, legwork, and/or creativity it takes to publish an article. In a presentation on its Web site, the MECC Envision mentions the “author dilemma: Who are they? Why are they authors? What is their role?” [51] All authors should ask the same questions of themselves.

## Abbreviations

CHC: Complete Healthcare Communications
CMD: Current Medical Directions
CRO: contract research organization
MECC: medical education and communication company
